# Racemose neurocysticercosis mimicking craniopharyngioma

**DOI:** 10.1590/0037-8682-0166-2025

**Published:** 2025-07-07

**Authors:** Cínthia Guedes Chaves, Nina Ventura, Diogo Goulart Corrêa

**Affiliations:** 1 Instituto Estadual do Cérebro, Departamento de Radiologia, Rio de Janeiro, RJ, Brasil.; 2 Universidade Federal do Rio de Janeiro, Departamento de Radiologia, Rio de Janeiro, RJ, Brasil.; 3 Universidade do Estado do Rio de Janeiro, Departamento de Diagnóstico por Imagem, Rio de Janeiro, RJ, Brasil.; 4 Clínica de Diagnóstico por Imagem (CDPI)/DASA, Departamento de Radiologia, Rio de Janeiro, RJ, Brasil.

**FIGURE 1:**
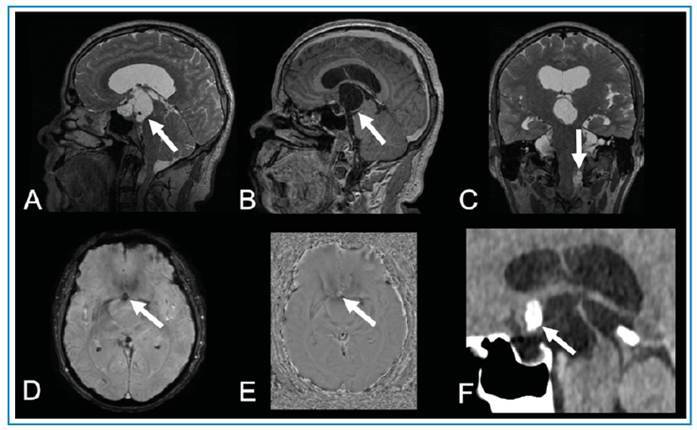
Suprasellar racemose neurocysticercosis mimicking craniopharyngioma. Brain MRI reveals a multiseptated suprasellar expansive cystic lesion on T2-weighted imaging **(arrow in A)**, with mild rim enhancement on T1-weighted imaging **(arrow in B)**, in the sagittal plane. A second extra-axial lesion can be observed in the left cerebellomedullary cistern on T2-weighted imaging in the coronal plane **(arrow in C)**. The suprasellar lesion presents a small calcic component, seen as a hypointense signal focus on susceptibility-weighted imaging (SWI) **(arrow in D)** and hyperintense signal focus on SWI phase imaging **(arrow in E)**, in a right-handed system. Brain CT confirms the lesion calcification **(arrow in F)**. Histopathological analysis confirmed the diagnosis of racemose neurocysticercosis.

A 52-year-old man presented with a 1-year history of generalized tonic-clonic seizures. Magnetic resonance imaging of the brain revealed a suprasellar multiseptated cystic lesion extending to the interpeduncular cistern and third ventricle, with nodular calcification and gadolinium enhancement at the posterior rim. A second extra-axial multiseptated cystic lesion was identified in the left cerebellomedullary cistern without gadolinium enhancement (Figure 1). The suprasellar cystic lesion was surgically removed because of the invasion of the third ventricle and the diagnostic hypothesis of craniopharyngioma. Histopathological analysis revealed that the cyst wall consisted of a cuticle layer and loose myxoid layer with lymphocytic infiltrates, confirming the diagnosis of racemose neurocysticercosis without a scolex. 

The most common suprasellar lesions are chiasmatic-hypothalamic gliomas, meningiomas, germinomas, craniopharyngiomas, and Langerhans cell histiocytosis (LCH). Suprasellar gliomas are low-grade solid lesions, without gadolinium enhancement. Typically, meningiomas are solid lesions with homogeneous enhancement. Germinomas usually present hypointense T2 signal and contrast enhancement. LCH presents as pituitary stalk thickening. Adamantinomatous craniopharyngiomas are solid cystic lesions with enhancement in their solid parts, and 90% of cases have calcifications^1^.

Neurocysticercosis is an infectious disease of the central nervous system caused by the larval form of *Taenia solium* (cysticerci) that occurs after egg ingestion. The infection progresses through four stages (vesicular, colloidal vesicular, granular nodular, and nodular calcified) and can be parenchymal, intraventricular, or subarachnoid^2^. Subarachnoid disease is referred to as the racemose form^3^ and is characterized by neuroimaging as an extra-axial cluster of cysts, often located in the basal cisterns or Sylvian fissures, without a visible scolex and with little or no edema^2^ ^,^ ^3^. In our case, the presence of another multiseptated cystic lesion was an indication for presurgical diagnosis.
